# 

**Published:** 1917-02-17

**Authors:** 


### HOSPITAL RESULTS.

**Pearson's Fresh-air Fund.**—The motto of the Fund is, "No one can be perfectly happy till all are happy," and the twenty-fifth annual report claims that the organisation has made 3,739,631 children happy. The accounts are dated November 7 and refer, a little vaguely, to "Summer, 1916"; the receipts for this unknown period were £10,155, and the expenditure £9,522. Children given a day's outing numbered 120,345, and those afforded a fortnight's holiday 4,192.

**King Edward VII. Memorial District Nursing Association, Halifax** (in affiliation with Queen Victoria's Jubilee Institute for Nurses).—The accounts and statistics are taken up to November 30, 1916. The receipts for twelve months were £788, and the expenditure £899. 13,590 visits were paid to 478 patients, 46 of whom were operation cases.

**Midland Hospital, Graaf Reinet, South Africa.**—Owing to the war, progress with new hospital buildings has been suspended. The building fund now stands at £3,967. During 1915, 354 in-patients and 2,969 out-patients were treated; 93 of those admitted were paying cases. Receipts, £3,562; expenditure, £3,231. The accounts are kept on the Uniform System.

**The Royal Salop Infirmary, Shrewsbury.**—This is one of the few larger hospitals in this country which arranges its accounts and presents its report to cover a period differing from the natural calendar year. In this case the institutional year ended on June 30, 1916, and the expenditure exceeded the income by £1,669, but the financial position is good on account of credit balances carried forward from previous years. Since the commencement of the war 737 sick and wounded soldiers have been treated at the infirmary, 485 of whom were admitted and 252 attended in the *x*-ray department for diagnosis and treatment.

**Salop County Asylum.**—In order to free accommodation for wounded soldiers 145 patients have been taken from the Rubery Hill Asylum, Birmingham, making the total number of patients in the charge of Salop Asylum 864 on the date when the latest count was made for the purposes of the report. Particulars are given of the percentage increase in average cost of food, and the following instances may be quoted: Brown sugar, 202 per cent.; beef, 128 per cent.; mutton, 98 per cent. The dinner served on the day when the Commissioner of the Board of Control visited the asylum consisted of liver and bacon with haricot beans and potatoes, followed by currant and boiled rice pudding.

**Leeds General Infirmary.**—One of the earliest returns of the complete financial business of 1916 comes from the General Infirmary at Leeds. The printed details of the year's work set forth not only the income and expenditure account, but a table showing the weight and cost of each principal article of food, drugs, dressings, wines, and spirits, etc., used. We have also been favoured with an elaborate table going even beyond this degree of thoroughness, in which one is able to discover the cost of maintaining each ward and department. Such a return is most valuable, in so far as it enables the administration to bring forcibly to the notice of the responsible officer an unusual rise in the outlay of any particular service. We notice that while the extraordinary income of the institution has remained stationary, the ordinary receipts have risen from £27,810 in 1915 to £34,836 in 1916. Contributions from workpeople outside the City of Leeds have increased from £1,893 to £3,321, and the War Office payments for wounded soldiers were £4,960, against £1,638. The total ordinary expenditure has risen from £42,746 to £50,453, and we are sorry to see that the authorities had to sell stock during 1916 to the value of £21,181 to provide funds for maintenance.

Rates of Subscription: Twelve months, 6/6; Six months, 4/-; Three months, 2/-; post free to any address in the United Kingdom. Abroad, Twelve months, 8/8; Six months, 5/-; Three months, 2/6, post free. The Hospital "Index" to Volume LX., April to September 1916, is now ready, price 2½d. per copy, post free. Address The Manager, "The Hospital," The Hospital Building, 28/29 Southampton Street, Strand, London, W.C.

